# Pruning chemicals from the green building landscape

**DOI:** 10.1038/s41370-019-0174-x

**Published:** 2019-10-07

**Authors:** Lisa J. Goodwin Robbins, Kathryn M. Rodgers, Bill Walsh, Rachelle Ain, Robin E. Dodson

**Affiliations:** 1 Kalin Associates, 1121 Washington Street, Newton, MA USA; 2 Silent Spring Institute, 320 Nevada Street, Newton, MA USA; 3 Healthy Building Network, 1710 Connecticut Ave NW, Washington, DC USA; 4 Bruner/Cott Architects, 225 Friend Street, Suite 701, Boston, MA USA

**Keywords:** flame retardants, perfluorinated chemicals, phthalates, inhalation exposure, volatile organic compounds

## Abstract

Green building design has substantially minimized environmental impacts by reducing energy consumption compared with traditional buildings. Yet, it is not uncommon for a green building to meet the highest criteria for energy efficiency and be built with materials that contain chemicals hazardous to occupant health. Because of this discrepancy in achieving holistic sustainability, the architecture/engineering/construction (AEC) industry has never been more interested in occupant health and well-being than it is today. At the same time, numerous scientific studies have documented exposures to and associated health effects of chemicals used in building materials. Opportunities to translate environmental health research so that it is useful to the AEC community exist across the landscape of healthier buildings. For example, research can be conducted to prioritize building material and chemical combinations to demonstrate how green building certification systems, government building codes, and the building products marketplace can increase energy performance while also addressing the greatest chemical exposures and health impacts. In order for scientific research to be used to create and support healthier environments, researchers should design and translate their research with this landscape in mind and should consider experts in the AEC industry as ambassadors for change. We provide key examples of how scientists have promoted healthy building practices and highlight additional research opportunities.

## Introduction

Green building is a nearly $100 billion industry in the United States [1] with direct impacts on reducing carbon emissions and indirect impacts on improving health. Recent estimates indicate that green buildings in the United States averted over 30 kilotons of CO_2_ emissions and conferred $2.7 billion in health benefits from reduced air pollutant emissions from electricity and fuel use from 2000 to 2016 [2]. Buildings account for approximately a quarter of greenhouse gas emissions globally, and the green building industry is projected to grow substantially to meet global development needs [3]. However, the mainstream green building industry focuses on resource efficiencies such as energy and water, missing opportunities to address exposures to harmful chemicals in building materials, which may have significant impacts on occupant health [4]. Under current conditions, it is not uncommon for a building to meet the highest criteria for energy efficiency, yet be built with materials that contain chemicals associated with cancer, neurotoxicity, and reproductive and developmental toxicity. Here is an opportunity to leverage substantial investments in green buildings to create healthier buildings by reducing harmful chemical exposures.

To make green buildings healthier, additional research is required on chemical hazards, indoor exposures, and occupant health. This investment in chemical research is neither required nor incentivized by the current US regulatory framework, which considers chemicals on an individual basis and often after serious harm to health is discovered [5]. However, there is a growing movement toward incorporating chemical guidance for healthier buildings within the green building industry. As this healthy building landscape develops, exposure scientists can develop evidence to prioritize chemical exposures with the biggest impacts on human health and provide evidence to support architects and builders’ decision-making about materials.

Lessons from past public health disasters provide ample evidence that not incorporating toxicology and exposure science into building decisions can have enormous costs to public health. Many retrospective studies have quantified the irreversible health, societal, and economic costs of lead in household paint used from the 1950s until it was banned in 1978 [6], leading to an estimated $953 billion in lead-based paint removal costs from US homes [7]. The use of polybrominated diphenyl ether (PBDE) flame retardant chemicals in upholstered furniture led to widespread exposures in the US, and a health and economic analysis estimated 11 million IQ points lost and an associated cost of $266 billion in 2010 [8]. Although structural similarities that PBDEs share with polychlorinated biphenyls, which were banned in the US in 1978, could have predicted health concerns decades ago, they were put into widespread use [9]. We are not destined to repeat these public health tragedies; researchers can work with the architecture/engineering/construction (AEC) community to integrate information for other hazardous chemicals used in building materials or products into best practices. For example, phthalates (used in vinyl flooring) are associated with disrupted testosterone production *in utero*, causing adverse male reproductive development [10]. Organohalogen flame retardant chemicals (used in insulation) are associated with cancer, thyroid disease, impaired cognition, and harm to reproductive systems [11–15]. Per- and poly-fluoroalkyl substances (PFAS) (used in paints, carpeting, and textiles) are associated with suppressed immune functioning, thyroid disruption, cancer, and many other adverse outcomes [16–19]. Scientific studies can identify effective strategies to reduce building occupant exposures to many commonly used toxic chemicals in order to avert future public health failures, and this knowledge needs to be translated for immediate and widespread use in the building industry.

**Fig. 1:**
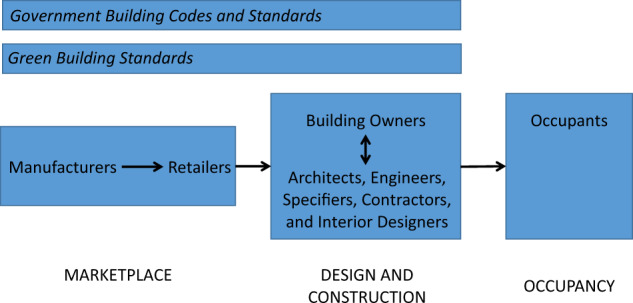
Healthy building landscape. Key phases in the development of healthier buildings that are opportunities for environmental health scientists to integrate research

Opportunities for research translation exist across the landscape of healthy buildings, which includes green building certification systems; government building codes and standards; product manufacturers and retailers; building owners; architects, engineers, specifiers, contractors, and interior designers; and occupants (Fig. 1). In this Perspective article, we discuss the impact that environmental health research has already had on the healthier building industry, and we provide examples at each phase in the landscape of healthier buildings. We also highlight remaining gaps that could be addressed with additional research. We focus on occupants’ chemical exposures from building materials, which have received some research attention but are emerging as a concern within the healthier building industry. Our aim is to motivate researchers to collaborate with architects and others in the building industry to infuse important research on chemical exposures into building design and construction, and to design studies that demonstrate the effectiveness of designing for healthier indoor environments.

## Green building certification systems

The US Green Building Council’s (USGBC) Leadership in Energy and Environmental Design (LEED) is recognized as the standard certification system for sustainable, high-performance buildings [20]. To achieve certification levels, prerequisite or optional credits are earned across different categories, such as energy and atmosphere and water efficiency. The impact of LEED on the built environment is far greater than the number of certifications. Municipalities have established LEED requirements in their building codes, sometimes without requiring full certifications and sometimes with adaptations to local climate and resources [21]. While LEED does have a Materials and Resources category with optional credits for building material transparency and hazard reduction, building projects are just beginning to achieve these credits as manufacturers begin to offer transparency information; though this credit’s uptake is hampered by limited knowledge about healthier materials within the AEC community. A review of certification systems worldwide revealed that the average contribution of indoor air quality (IAQ) to these systems is only 7.5%, and that IAQ is often managed through ventilation (effective for only some indoor pollutants) and sometimes emission controls and indoor air testing [22]. LEED’s main competitor, Green Globes [23], has optional credits that address life cycle analysis of building materials, but not material health. Newer certification systems, such as the Living Building Challenge (LBC) [24], WELL [25], and Fitwel [26], more explicitly address building materials and associated hazards, for example through red lists of chemicals to be avoided (Table 1).

**Table 1:** Major green building certification systems in the United States with criteria for chemicals in building materials

Certification name	Supporting organization	Start date	Number of certifications in US	Materials and human health criteria
Leadership in Energy and Environmental Design (LEED)	US Green Building Council	1998	80,000+	Offers optional material health safety measures for building product disclosure and optimization through Health Product Declarations, Cradle to Cradle certifications, and Declare product labels; for materials with low VOC emissions, meeting CDPH MSV1.1-2010, Section 8^a^, with third party certification; and for pilot credits that minimize the use of mercury, lead and cadmium
Green Globes	Green Building Initiative	2004	1600	Offers optional criteria materials with low VOC emissions, meeting CDPH SM V1.1-2010, Section 8^a^, with third-party certification
Living Building Challenge (LBC)	International Living Future Institute	2006	33	Requires elimination of Red List chemicals in 90% of a project’s new material by cost; pre-existing materials are exempt. Red List: *Antimicrobials (marketed with a health claim), Alkylphenols and related compounds, Asbestos compounds, Bisphenol A (BPA) and structural analogs, California-banned solvents, Chlorinated Polymers (including Chlorinated polyethylene (CPE), Chlorinated polyvinyl chloride (CPVC), Chloroprene (neoprene monomer), Chlorosulfonated polyethylene (CSPE), Polyvinylidene chloride (PVDC), Polyvinyl chloride (PVC)), Chlorobenzenes, Chlorofluorocarbons (CFC) and hydrochlorofluorocarbons (HCFC), Formaldehyde (added), Monomeric, polymeric and organophosphate halogenated flame retardants (HFR’s), Organotin Compounds, Perfluorinated compounds (PFC’s), Phthalates (orthophthalates), Polychlorinated biphenyls (PCB’s), Polycyclic aromatic hydrocarbons (PAH’s), Short-chain and medium-chain chlorinated paraffins, Toxic heavy metals (Arsenic, Cadmium, Chromium, Lead (added), Mercury), Wood Treatments containing creosote or pentachlorophenol* Volatile organic compounds (VOC) (wet-applied products) are limited but not banned.
WELL	International Well Building Institute	2014	159	Offers optional material health safety measures through elimination or restriction specified chemical classes^b^: *PFCs, HFRs, phthalates, isocyanate-based polyurethane, and urea-formaldehyde* or through elimination of LBC red list chemicals (see above) in at least 25% of materials or at least 25% of the products meet third-party hazard certifications, including LBC, Cradle to Cradle, and GreenScreen certifications.^c^
Fitwel	Center for Active Design	2017	180	Offers optional material health safety measures through green purchasing policies, including US EPA Safer Choice Label, UL ECOLOGO, and Global Ecolabelling Network. There are no requirements for transparency or red list-free materials.

^a^California Department of Public Health Standard Method for the Testing and Evaluation of Volatile Organic Chemical Emissions from Indoor Sources using Environmental Chambers Version 1.1, 2010

^b^Toxic material reduction (Feature 25)

^c^Enhanced material safety (Feature 26)

Exposure scientists and toxicologists play a critical role in the design of certifications programs aimed at reducing exposure to hazardous chemicals in building materials. The LBC Materials Petal is the most rigorous of these certification systems. Creators of the Materials Petal prioritized chemicals with potential for widespread exposure, chemicals that are bioaccumulative, and chemicals that may pose a risk to factory and construction workers [27]. The LBC “Red List” includes individual chemicals such as chromium VI and bisphenol A, classes of chemicals such as halogenated flame retardants and perfluorinated chemicals, and specific materials such as polyvinyl chloride (PVC) [28]. The Red List is updated with every release of LBC; the count of prohibited chemicals, as of LBC v3.1, was 817.

Two additional building certifications that have emerged in the past few years that focus on human health and wellness are the WELL Building Standard, administered by the International WELL Building Institute, and Fitwel, administered by the Center for Active Design on behalf of the US Department of Health and Human Services. Both standards address human health through IAQ, daily physical activity, proximity to public transit and fresh food, and are based on peer-reviewed scientific research. While neither of these standards meets the high bar set by LBC’s Materials Petal for elimination of the most hazardous chemicals, each offers valuable platforms for the AEC industry to improve building environments and for exposure scientists to engage and collaborate. While the current WELL standard platform is best applied to workplace environments, WELL is producing pilot programs for housing, institutional, and other types of buildings. Fitwel offers a certification that can be applied more broadly to meet its creators’ goal for every building to come with a Fitwel score.

These newer certification systems focus on human health and wellness, supporting the research links between occupant well-being, productivity, cognitive function, and healthy indoor environments [29], which are compelling incentives for both building owners and business leadership [30, 31]. Their profitability and bottom lines depend on the well-being of people who live and work in their buildings. As such, these certification programs have the potential to become widespread, raise the bar on indoor environment quality, and provide information to building owners, facility operations personnel, and occupants. Harmonization—alignment in reporting requirements—across the certifications will help to increase their uptake.

Environmental health scientists can support stronger and more effective building certification systems by engaging with programs as experts and by designing research studies to evaluate the efficacy of certification programs, particularly where harmonization across programs exist, to reduce indoor chemical exposures and improve health. There have been few studies to date that have compared exposures and reported health in green and conventional buildings. For example, green subsidized housing units in Boston, MA had significantly lower PM_2.5_ and NO_2_ concentrations compared with control units, and children living in green buildings experienced lower risk of asthma symptoms compared to those children not living in green buildings [32, 33]. However, the green buildings mostly focused on energy efficiencies and much of the benefits (lower pollutant levels and better self-reported health) may be attributable to integrated pest management policies and improved area ventilation. Other studies have mostly focused on occupant surveys and not chemical measurements [34]. Rather, exposure studies in green certified buildings that account for material hazards (e.g., LBC-certified building) compared to green certified buildings that do not consider material choices can quantify the impact of the material selection on exposures and inferred improvements on occupant health. Also, more studies are needed to examine the health effects of simultaneous exposures, since indoor exposures are chemical mixtures.

## Government building codes and standards

Building codes and standards for building materials influence chemical exposures. Some may directly target chemicals, such as California Air Resources Board’s regulations on formaldehyde emissions from particleboard and composite wood products [35]. Other performance-based standards may be created with efficiency or safety in mind, but their limited consideration for associated chemical exposures and their impact on health needs to be addressed. For example, building insulation codes and furniture flammability standards in California have led to widespread use and exposures to flame-retardant chemicals. The International Residential Code, used in 49 US states [36], does not address chemical use, and neither do most, if not any, local building codes that require more energy efficient practices.

Exposure scientists have the ability to generate and translate information that can lead to safer and healthier codes and standards. For example, exposure studies that documented elevated flame retardant exposures associated with furniture meeting California’s flammability standards for residential [37] and public use [38] contributed to California’s revision of its flammability standards to achieve fire safety without compelling the use of added flame retardants [39, 40]. Changes to the fire codes in Massachusetts and Boston followed [41, 42], and many states have enacted legislation to ban flame retardants from furniture, bedding, and/or baby products [43]. Further, these data were critical to the decision by the US Consumer Product Safety Commission to initiate rulemaking on a ban of organohalogen flame retardants in furniture, mattresses, electronics, and children’s products [44]. Similarly, California recently changed the state building code to allow polystyrene plastic insulation used below grade to be free of flame retardants [45] because of research on the harms and lack of safety benefits of flame retardants in these circumstances [46].

A benefit of code, standard, and policy changes related to chemical use is that these changes can spur innovation and health-based improvements in products. For example, California’s standards for formaldehyde emissions from composite wood products solidified a market demand to develop formaldehyde-free materials. If exposure scientists design studies to demonstrate the effectiveness of certain laws or codes in lowering chemical exposures, that data can help energize movements to implement those rules in other places and at larger scales. These studies should be accompanied by education for manufacturers, testimony to legislative bodies, and support for policy changes.

## Marketplace

The building material marketplace is evolving to meet the requirements of the green building certification systems. This includes beginning efforts to increase transparency in the marketplace where manufacturers and retailers disclose material ingredients, which are often compared to hazard lists. The development of hazard lists also has an influence on the market as manufacturers compete to create products free of hazardous chemicals. Much of the focus, however, has been on traditional IAQ pollutants, such as volatile organic compounds (VOCs), while there are many other important chemical hazards that do not receive attention, such as semivolatile organic compounds (SVOCs). Programs that incorporate information on chemical hazards with exposure are also being developed, and these programs will push key sectors of the building material market to innovate by ranking relative risks of building materials. Through evaluating hazards, developing exposure scenarios, and defining the scope of the chemicals of concern in building materials, exposure science has and should play a critical role in the evolving marketplace.

### Product content inventory

Unlike food products, which must disclose their ingredients by the US law [47], the chemical contents of building products are not required to be disclosed. Products that claim to be free of “red list” chemicals, often are not forthcoming about which, if any, chemicals are being used instead, raising concern about regrettable substitution. Product testing studies have helped to better understand chemical contents. For example, testing in 2015 found that 58% of 65 vinyl flooring sample products sold by national home improvement retailers contained ortho-phthalates [48], which spurred a pledge by retailers to prohibit use of ortho-phthalates by their vinyl floor vendors. Subsequent testing in 2019 found no ortho-phthalates in random samples purchased at national retail chains [49].

However, independent product testing and IAQ testing are expensive. An essential step to characterizing chemical exposures in the built environment is transparency: complete disclosure of product contents in a standard format. Reliable inventory information is increasingly becoming available through the Health Product Declaration (HPD), a voluntary open standard format for the disclosure of product content information that accounts for among other things, the concentration (in ppm) of chemical constituents, the role of the chemical in the product, and associated health hazard [50]. HPDs are available in the “public repository” of the HPD Collaborative, a not-for-profit member association, representing the full spectrum of the building industry. However, the 4400 published HPDs address only a fraction of the tens of thousands of building product variants. To fill this gap, the Healthy Building Network (HBN) has compiled 100 “Common Product” profiles that list chemical contents from HPD reported data and other publicly available sources for the most commonly used substances (vinyl composition tile, for example) [50, 51]. HPDs and common product profiles can be used as tools by researchers to help design and target exposure testing. For example, knowing the chemical levels in commonly used building products, an exposure scientist could test for chemicals in spaces that have specified products free of those chemicals at varying degrees in order to prioritize products from an exposure and health perspective. HPDs also permit review of safety data for the chemicals in the product.

### Chemical hazard assessment

The marketplace is creating research-based tools for architects, building developers, and product developers to know what health hazards are associated with chemical constituents in building products. Chemical hazard assessments, a systematic review of the toxicological literature available on a chemical, are recognized as foundational tools for building material health hazard and risk assessment, alternatives comparison, and product innovation. The GreenScreen for Safer Chemicals [52] is a chemical-specific scoring tool for hazard assessment and safer alternative selection that relies on over 40 authoritative hazard lists including European Union’s Registration, Evaluation, Authorisation and Restriction of Chemicals  (REACH) categorizations and chemical hazard classifications by countries using the Global Harmonized System of Classification and Labeling of Chemicals [52]. Each GreenScreen Specified List is mapped to hazard endpoints and a hazard level or range based on GreenScreen Hazard Criteria. GreenScreen information is reported in the HPD Open Standard to communicate ingredient hazard.

Exposure scientists may want access to chemical hazard information in order to prioritize analytical targets for a study design; knowledge of commonly used chemicals in building materials along with chemicals with the highest health concerns can inform study designs to test exposure reduction strategies. However, relatively few chemical hazard assessments are currently publicly available. Those that are can be accessed through HBN’s Pharos Project database (for a subscription fee) [51]. Pharos also includes a GreenScreen List Translator function, which automatically screens chemicals against GreenScreen’s Specified Lists generating GreenScreen Benchmark Scores. While not as comprehensive as the full GreenScreen assessments, the List Translator can provide exposure scientists with reliable information for study design.

To address the lack of full chemical hazard assessments that are necessary for thorough comparisons of chemical alternatives, a new initiative known as MaterialWise [53] is being developed. MarerialWise, which draws upon chemical screening data from Pharos, is an innovative precompetitive value chain collaboration focused on increasing manufacturer access to high-quality third party-verified toxicological assessments [53], while making this information more affordable and actionable. Increasing availability of hazard assessments will create additional research opportunities for exposure scientists for targeted research on chemicals of the most concern.

### Limited exposure scenarios

Current exposure research on VOCs and SVOCs can inform product and building certifications. Although the building industry has made great strides in reducing VOC emissions in buildings by incorporating many low-VOC certifications into standards such as LEED, Green Globes, LBC, WELL, and Fitwel, further challenges remain [22]. For example, a 2013 study identified a significant number of VOCs associated with asthma that are in building products but that are not adequately included in leading product certification standards [54].

Greater challenges remain in addressing SVOCs. Whereas VOC releases from building materials can be readily modeled and measured, with releases that tend to be higher from new building products and decline over time, the dynamics of SVOCs indoors are more complicated due to their potential to partition readily onto surfaces and redistribute overtime [55, 56]. SVOCs also tend to be released over longer periods of time, and emission rates can vary due to conditions in the indoor space, including ventilation rates, temperature, humidity, and particulate matter loading [57–59]. SVOCs are among top priorities facing the building material industry today. These include phthalates from vinyl floor and wall coverings [60]; PFAS used as stain and water repellents on carpets and upholstery [61, 62]; and flame retardant chemicals added to insulation, upholstered furniture, and textiles [63]. New concerns about heavy metal contamination have arisen with the increase of unregulated recycled content, including fly ash, crumb rubber, and PVC plastic, in many building products [64–66].

Building product certifications and standards that address chemical emissions mostly focus on potential exposures to the occupant. Exposure studies are also needed in the construction and manufacturing workforce, as well as fence-line communities adjacent to building product manufacturers and construction waste disposal sites [67–70]. These areas are often under-represented in life cycle analyses supporting material specifications. This is an essential step in ensuring environmental equity and social justice in order to guard against emissions reduction strategies (such as factory and construction site off-gassing) that potentially shift risk to vulnerable communities.

### Moving the market

Transparency (i.e., ingredient disclosure) and the hazard evaluation of building materials has created market-based opportunities to develop new building materials that meet the requirements of the various green building standards. However, the landscape of product innovation is vast. To accelerate product innovation throughout entire building product categories, HBN is developing Transformation Targets for product categories that combine hazard information with exposure data to prioritize specific product categories. Exposure data include information on chemical use within a product, total volume of a chemical within a product category, and, in some cases, information from available occupational and residential studies [71]. Such exposure data led to the prioritization of phthalates in flooring, wall coverings, and furniture; bisphenol A in coatings, sealants, adhesives, and grouts; flame retardants in building insulation, window treatments, and furniture; and fluorinated chemicals in carpets, wall coverings, window treatments, and upholstery. Exposure data are needed to prioritize additional chemical + product combinations.

Market-based initiatives have been used by HBN and the national grassroots consumer campaign Mind The Store [72] to successfully negotiate an agreement with Home Depot (soon followed by all major building products retailers) to eliminate 12 chemicals, including methylene chloride, phthalates, alkylphenol ethoxylates, and perfluorooctanoic acid (PFOA) and perfluorooctane sulfonate (PFOS), entirely from six product categories (paints, paint removers, flooring, insulation, cleaning, and gardening) [73]. Similar transformation target guidance is available in HBN’s HomeFree program that supports affordable housing efforts to improve human health by using less hazardous building materials [74].

## Design and construction

Building codes, green building certification systems, and the building material market come together in the design and construction of a new building. Therefore, the best time to select healthier materials for buildings is during the project’s design and construction, when building owners and the AEC community work together to make decisions that will be put in place for decades to come. Open communication between environmental health researchers and architects is critical to form decisions with long-lasting impacts.

The benefit of using building certifications that focus on overall occupant health, such as WELL and Fitwel, is that they engage the stakeholders—building owners, architects, and facilities management—in the design and construction processes. One of WELL’s requirements includes a Stakeholder Charrette during the design process; other requirements for certification include creating health-related policies and regularly scheduled maintenance and testing. This engagement provides the opportunity to educate and raise awareness about exposure science and materials beyond the standards’ requirements.

The design phase represents a particularly valuable opportunity to influence building material choices. Architects and designers often select and specify familiar and proven items that meet desired targets for performance, durability, cost, and aesthetics. When it comes to ubiquitous construction materials and products (e.g., gypsum board, metal studs, resilient flooring, adhesives, paints, and sealants), which comprise the most material in a building by volume and have a proportionally larger impact on occupant health, architects usually rely on materials from previous projects. There is little incentive to change as long as desired targets are met. Further, the AEC community primarily bases decisions on performance, while chemical information about products is usually sparse and comes directly from building product manufacturers, which is often rife with “greenwashing”—a practice of overstating environmental benefits. As a result, the latest environmental health science is not considered in the selection of building materials for the majority of building projects, representing a critical gap between environmental health research and design practice.

As health and exposure issues are coming to the fore in design, architects and designers need clear direction in making better choices. While there are many red lists detailing chemicals to avoid, there is very little information about finding better alternatives and avoiding “regrettable substitutions.” HPDs and GreenScreen provide information about the chemical content of products and materials and their associated health hazards; however, most designers need constructive guides to search for and choose viable alternate products. While Transformation Targets, prioritized product and chemical combinations, will help fill this gap, designers should collaborate with exposure scientists in order to make progress in the interim and across the entire building material landscape.

Exposure scientists need to share their latest research with the AEC community. Researchers should consider making presentations at conferences attended by the AEC community, like American Institute of Architects meetings, Construct, USGBC GreenBuild, International Living Futures Institute meetings, National Association of Homebuilders meetings, and NeoCon. These non-science design and construction specialists need guidance in how to select, specify, and procure better, less hazardous, building products for all projects, not just those seeking green building certifications. Further, when communicating directly with the AEC community, researchers should avoid jargon, focus on the larger concepts (e.g., general dynamics of pollutants indoors), and be consistent. The Six Classes of Chemicals, introduced by the Green Science Policy Institute, for example, provides a framing for talking about chemicals that is accessible to nonresearchers and can be applied across many areas of research [75].

There are examples in healthcare and affordable housing where research has been used to take action ahead of changes to building codes or green building standards thus demonstrating the potential impact of sharing environmental health research. In May 2015, Kaiser Permanente, citing research from the Centers for Disease Control and Prevention, GreenScreen data, and antimicrobial chemicals listed the Pharos Project, issued a design bulletin that prohibits the specification of products containing 13 antimicrobial chemicals and materials as part of comprehensive commitment to fighting antibiotic resistance [76]. In the affordable housing sector, the Housing Partnership Network, a membership organization of 100 leading affordable housing nonprofits that own over 250,000 rental units, is implementing procurement and transparency guidelines based upon HomeFree’s hazard spectrum guidance [77]. This will bring healthier building products to low income households who are disproportionately exposed.

Once educated, design professionals can make better choices, either through the selection of materials with safer chemical alternatives or specifying materials that do not contain hazardous chemicals. For example, easily cleanable multicolored resilient flooring could be selected instead of white carpet coated with PFAS. Similarly, architects should not overdesign spaces. Light commercial occupancies do not require the same high-performance chemical coatings as a research laboratory. These choices not only benefit future occupants but also subcontractors installing these materials.

## Occupancy

Indoor chemical exposures, and associated health impacts, result from chemical emissions from building materials as well as from the myriad of products introduced by the occupant. In other words, unwanted chemical exposures can occur even in the most diligently specified building. Teasing apart the relative impact of the building versus the occupant can help develop exposure reduction strategies and understanding appropriate points of intervention (e.g., green building standards versus occupant-oriented actions).

Most indoor exposure studies have been conducted during occupancy when the measured concentrations represent aggregation of sources, including building materials and consumer products brought in and used by the occupants. These studies reveal widespread exposures to commercial chemicals, including phthalates, flame retardant chemicals, and PFAS [78]. Identifying the sources of indoor contaminants is critical in order to develop effective exposure reduction strategies, including upstream product reformulation. Using a unique study design, researchers measured indoor air concentrations of nearly 100 VOCs and SVOCs in newly renovated subsidized housing units in Boston, MA before and after occupancy [79]. By comparing concentrations measured before occupants moved in and after they lived in the units for at least one month, chemicals were classified as having predominantly building sources (e.g., from building materials), predominantly occupant sources (e.g., from personal care products used by the occupants), or a combination of both building and occupant sources. Chemical categorization informs strategies to reduce exposures; those chemicals that come mostly from building sources can be targeted through material specification whereas those chemicals that come mostly from occupant sources can be targeted through occupant education programs. VOCs such as toluene and xylene had clear building-related sources, as expected. However, SVOCs such as benzophenone, a UV filter used in personal care products, dibutyl phthalate, a phthalate used in nail polishes, and tris(1-chloro-2-propyl) phosphate, a flame retardant, also appeared to be from building sources because they were detected both during pre- and post-occupancy sampling. These SVOCs are not within the scope of most green building certification systems.

Studies have shown that green buildings generally improve health and well-being of occupants [80], which is of great public health importance, especially when these improvements are experienced in disadvantaged communities overburdened with other environmental contributors to disease. However, much of the measured health improvements may be the result of improvements in thermal comfort and ventilation afforded by newly constructed green buildings. In addition to these short-term health gains, research is needed to quantify the impacts of material selection on chemical exposures and subsequent long-term health. For example, a study that compares exposures within two green certified buildings, one of which considered healthier material selection, can bolster the importance of material selection in building certification systems by providing the necessary evidence-base. Measuring impacts on health is more complex since many of the chemicals of interest are associated with chronic health conditions with subtle health impacts; however, impacts on health can be inferred based on differences in exposure.

## Conclusions

In order for green buildings to be truly sustainable and prioritize health, they need to move beyond resource efficiencies and indirect impacts on health. Healthy buildings comprehensively consider the impact of building materials on chemical exposures and occupant health. Exposure scientists play an important role in this transition to healthy buildings by conducting research on indoor chemical exposures. There are opportunities for researchers to study the efficacy of green certification programs to reduce exposures, to design studies that demonstrate the impact of building code or policies on exposure, to test and prioritize chemical and material combinations, to incorporate measures relevant to long-term health into exposure studies, and to share and translate the latest indoor environment science for implementation by the AEC community. As the evidence emerges, it is important that the AEC community consider critically whether hazardous chemicals are needed in specified building materials. All the while, mainstream green building standards and their supporting organizations need to continue to push for transparency and safer products in the marketplace.
